# Using Pre-Clinical Studies to Explore the Potential Clinical Uses of Exosomes Secreted from Induced Pluripotent Stem Cell-Derived Mesenchymal Stem cells

**DOI:** 10.1007/s13770-023-00557-6

**Published:** 2023-08-31

**Authors:** Andrew Kailin Zhou, Eric Jou, Victor Lu, James Zhang, Shirom Chabra, Joshua Abishek, Ethan Wong, Xianwei Zeng, Baoqiang Guo

**Affiliations:** 1 Addenbrookes Major Trauma Unit, Department of Trauma And Orthopaedics, Cambridge University Hospitals, Cambridge, UK; 2 School Of Clinical Medicine, University Of Cambridge, Cambridge, UK; 3 Department of Life Science, Faculty of Science and Engineering, Manchester Metropolitan University, Manchester, UK; 4 Beijing Rehabilitation Hospital Affiliated to National Research Centre for Rehabilitation Technical Aids, Ministry of Civil Affairs of China, Beijing, China; 5 Watford General Hospital, London, UK; 6 Weifang People’s Hospital, Weifang City, Shandong Province China

**Keywords:** MSC, Exosome, iPSC, Stem cell

## Abstract

Recent studies of exosomes derived from mesenchymal stem cells (MSCs) have indicated high potential clinical applications in many diseases. However, the limited source of MSCs impedes their clinical research and application. Most recently, induced pluripotent stem cells (iPSCs) have become a promising source of MSCs. Exosome therapy based on iPSC-derived MSCs (iMSCs) is a novel technique with much of its therapeutic potential untapped. Compared to MSCs, iMSCs have proved superior in cell proliferation, immunomodulation, generation of exosomes capable of controlling the microenvironment, and bioactive paracrine factor secretion, while also theoretically eliminating the dependence on immunosuppression drugs. The therapeutic effects of iMSC-derived exosomes are explored in many diseases and are best studied in wound healing, cardiovascular disease, and musculoskeletal pathology. It is pertinent clinicians have a strong understanding of stem cell therapy and the latest advances that will eventually translate into clinical practice. In this review, we discuss the various applications of exosomes derived from iMSCs in clinical medicine.

## Background

Mesenchymal stem cells (MSCs) are regarded as one of the most promising prospects in bio-engineering due to their multipotent ability and accessibility [1]. MSCs can differentiate into an extensive array of cell types such as adipocytes, osteoblasts, chondrocytes, cardiomyocytes, and neurons and have the potential to be utilised in a broad range of therapeutic applications [2]. However, MSCs transplantation may have associated risks, including potential tumour formation [3, 4]. Following infusions, MSCs have been shown to distribute in a wide range of organs without specificity; studies have reported cases in the lung, kidney, thymus, bone, skin, gastrointestinal tract, liver and bone marrow [5–7]. This may have implications in tumour formation. However, there seems to be a bidirectional relationship between MSCs therapy and cancer; the anti-tumour and tumour-forming effects are highly variable and dependent on the cells themselves [3].

In the past two decades, increasing studies have identified MSC-derived extracellular vesicles (EV) to be responsible for the therapeutic effects of the MSCs [8, 9]. EVs include exosomes, microvesicles and apoptotic bodies, and they function as intercellular messengers secreted by cells to aid in cell signalling [10]. In particular, exosomes contain a wide range of biologically active molecules, including growth factors, cytokines, mRNAs and regulatory RNAs, and serve as delivery machinery that exerts paracrine effects on nearby cells [11]. Structurally, exosomes consist of a lipid bilayer with a hydrodynamic radius of 50-100 nm, and this bilayer plays a significant role in maintaining the stability of the proteins and nucleic acids inside [9]. Furthermore, EVs are believed to play a crucial role in cellular communication [1]. Recently, there is increasing appeal in using MSC-derived EVs as a therapeutic alternative to MSCs [11], the rationale being that exosome-based therapies may mitigate the safety concerns associated with the use of MSCs such as arrhythmia in the heart, tumorigenesis, ossification, infusion toxicities, and calcification in tissues [12, 13].

MSCs are commonly obtained from tissues such as bone marrow, umbilical cord blood and fat tissue and therefore they are called adult MSCs. An alternative source of obtaining MSCs is induced pluripotent stem cells (iPSCs), which provide patient-specific adult somatic cells while showing similar capabilities and morphology to embryonic stem cells (ESCs) in terms of self-renewal and differentiation [14, 15].

MSCs obtained from iPSCs (iMSCs) can meet the unmet clinical needs through their inexhaustibility [16]. The main benefit of iMSCs is that they do not have to be autologous as they are free of HLA-DR expression [17]. Allogenic iMSCs are compatible with the world population, making them an attractive future clinical therapy for a variety of pathologies.

Compared to adult MSCs, iMSCs have proved superior in cell proliferation, immunomodulation, generation of exosomes capable of controlling the microenvironment, and bioactive paracrine factor secretion, while also theoretically eliminating the need for immunosuppression [18].

Further benefits of iMSCs therapy over adult MSCs therapy are listed below:

1. Bloor et al. found that one iPSC bank can produce 29 million clinical doses of iMSCs therapy [17]. iPSCs are considered an inexhaustible source of iMSCs that can meet the high clinical demand [16].
2. iMSCs are superior to adult MSCs regarding the generation of exosomes, cellular proliferation, immunomodulation, bioactive paracrine factor secretion and microenvironment modulation [18].
  - Cellular proliferation: the therapeutic effects of bone marrow-derived MSCs (BM-MSCs) is limited through their limited proliferative potential [19]. iMSCs exhibit greater proliferation potential than traditional adult MSCs [20].
  - Immunomodulation: *In vitro,* iMSCs are just as effective as BM-MSCs at down-regulating NK cytolytic capabilities. Moreover, iMSCs are more impervious to being destroyed by preactivated NK cells when compared to BM-MSCs [21]
  - Factor secretion: iMSCs possess many of the beneficial biological effects that adult MSCs exert, but are also shown to be superior in factor excretion [18, 21].
  - Microenvironment-modulating exosomes: iMSCs are superior to adult MSCs in producing microenvironment-modulating exosomes [18, 21].
3. Ethics: iPSCs hold a similar self-renew capability to ESCs but are free of ethical issues [15].
4. Virus transmission: adult MSCs have a far greater risk of virus transmission than iPSCs-derived MSCs [22, 23].

The therapeutic effect of iMSCs-derived EVs are explored in many diseases and are best studied in wound healing, cardiovascular disease and musculoskeletal pathology [16, 24, 25]. In this review, we discuss the various applications of exosomes derived from iMSCs in modern medicine (Table 1).

**Table 1:** *In vivo* therapeutic applications of iMSCs-derived exosomes in animal disease models

Disease type	Model	Main outcome	Mechanism	References
Osteoarthritis	Osteoarthritis mouse model	A greater therapeutic effect was observed in iMSC-Exos when compared to SMMSC-Exos	Chondrocyte proliferation was stimulated to a more substantial effect in iMSC than SM-MSCs	Zhu et al. [16]
Osteoporosis	Engineered rat tissue	Exosome and tricalcium phosphate combination form a scaffold which promotes bone regeneration	This combinatorial scaffold changed the expression of genes involved in the PI3/Akt pathway, resulting in osteogenesis	Zhang et al. [43]
Osteoporosis	Bone defect model in ovariectomised rats	Observed attenuation of osteoporosis with iMSC-Exos	iMSC-Exos stimulated osteogenesis and angiogenesis	Qi et al. [41]
Osteoporosis	Steroid-induced osteonecrosis of the femoral head in rats	Preventing osteonecrosis of femoral head through angiogenesis	iMSC-Exos were responsible for activation of PI3/Akt pathway on endothelial cells	Liu et al. [44]
Osteonecrosis	Steroid-induced osteonecrosis of the femoral head in rats	Alleviate severity of osteonecrosis of femoral head by preventing osteoblast apoptosis	miR-135b secreted from iMSC-Exos inhibits expression of PDCD4.	Zhang et al. [115]
Myocardial ischaemia	Swine myocardial ischaemia model	Improves physiological cardiac function recovery in a myocardial ischaemia swine model	Exosomes reduced apoptosis, maintained intracellular calcium homeostasis, and raised adenosine 5'-triphosphate	Gao et al. [53]
Myocardial ischaemia	Mouse myocardial infarction model	Promotes autophagy of hypoxic cardiac myocytes in the recovery of myocardial ischaemia	The predominant pathways regulating autophagy were PI3K‐Akt‐mTOR, insulin, and MAPK signalling pathways	Santoso et al. [52]
Myocardial Ischaemia	Rat model of Severe Acute Pancreatitis (SAP) induced Myocardial Ischaemia	Improved cardiac function and reduced oxidative stress after SAP-induced myocardial ischaemia	Inhibition of Nrf2/HO-1 resulted in improved LVDs and LVDd. Established the importance of the Akt/Nrf2/HO-1 signaling pathway in preventing adverse cardiac outcomes post-SAP	Chen et al. [113]
Limb ischaemia	Mouse ischaemia model	Inhibits limbs ischaemia by promoting angiogenesis	iPSC-EVs containing miR-199b-5p dramatically increased microvessel density and blood perfusion	Hu et al. [106] Ye et al. [107]
Skin wound	Rat wound healing model	Promotes re-epithelisation, collagen maturity and reduces scar widths	Transplanting iMSCs-Exos subcutaneously accelerated proliferation and migration of human dermal fibroblasts	Zhang et al. [25]
Diabetic wound ulcer	Diabetic ulcer mouse model	Quicker wound closure, increased density of blood vessels and nerve fibers	iMSC-Exos significantly promoted the migration of fibroblasts to wound sites compared to the control	Kobayashi et al. [57]
Skin wound	Rhesus macaque wound healing model	Increased wound healing in autologous iPSC exosome therapy compared to allogenic iPSC exosome therapy	iPSC-Exos accelerated wound closure, epithelial coverage, collagen deposition, and angiogenesis	Lu et al. [64]
Rett’s syndrome	Mouse dentate gyrus model	Exosomes can regulate neural circuits–treatment can increase proliferation and differentiation of neurones *In vitro*. Also observed an increase in proliferation of granule cell layer in dentate gyrus	Proteomic and bioinformatic analysis revealed exosomes can reverse the phenotype in MECP2* mutant neurons	Sharma et al. [78]
Eye	Rat corneal defect model	iMSC exosome therapy promotes more effective healing compared to MSC exosomes	Upregulation of CDK2 and cyclin A to catalyse corneal epithelial cells to enter the S phase of the cell cycle	Wang et al. [84]
Eye	Rat corneal injury model	iMSC-exosomes combined with a thermosensitive hydrogel, reduce scar formation and accelerate wound healing.	Downregulation of collagen expression in the corneal stroma. Exosomes containing miR-432-5p prevent ECM deposition via TRAM2 suppression.	Tang et al. [116]
End-stage kidney disease	Unilateral ureteral obstruction mouse model	Reduction in renal fibrosis and improved renal function	Reduction in the differentiation of NRK-52E cells	Liu et al. [91]
Acute Kidney Injury	Murine renal ischaemia/reperfusion injury model	Improved cell growth and survival after renal ischaemia/reperfusion	Increased activation of the ERK1/2 phosphorylation signalling pathway.	Lim et al. [112]
Cirrhosis	Murine hepatic ischaemia/ reperfusion injury model	Prevent further hepatic ischemia/ reperfusion injury	Activates SK1* and S1P1* signalling pathway	Du et al. [92]
Cirrhosis	Rat hepatic ischemia/ reperfusion injury model	Prevent further hepatic ischemia/ reperfusion injury	1. Anti-inflammation (Tumour necrosis factor-alpha, IL-6*) 2. Anti-apoptosis (Caspase-3, bax) 3. Anti-oxidation (glutathione, glutathione Peroxidase, SOD*)	Nong et al. [93]
Premature Ovarian Failure	POI-like mouse model induced by chemotherapy drugs	Preservation of ovarian function, and slowing of follicle loss	Inhibition of granulosa cell apoptosis via upregulation of NRF2 gene expression, leading to anti-oxidation via SOD1 and GCLC.	Zhang et al. [114]

## Exosomes

As aforementioned, exosomes play a vital role in intercellular communication by containing and conveying integral biologically active molecules, which change the activity of target cells through a number of different methods [11]. Depending on the cell of origin, EVs, including exosomes, can contain many constituents of a cell, including DNA, RNA, lipids, metabolites, and cytosolic and cell-surface proteins [26].

Synthesis of exosomes involves a process that first begins with double invagination of the cells plasma membrane, resulting in the production of multivesicular bodies (MVBs) within the cell. The first stage of this double invagination process leads to initial formation of a cup like structure that eventually envelops surface and extracellular proteins, causing the production of an early sorting endosome. The early sorting endosome goes through several steps of development (with formation of a late sorting endosome as an intermediate), finally generating MVBs. These so-called MVBs are formed after a second invagination within the endosome, giving rise to intraluminal vesicles (ILVs), which are resultantly released from the cell through membrane fusion and exocytosis [26].

Extracellular vesicles, including exosomes, can be extricated using a variety of different methods. The 6 commonest in the literature have described to be: differential ultracentrifugation (dUC), size-exclusion chromatography (SEC), ultrafiltration (UF), polyethylene glycol-based precipitation (PEG), immunoaffinity capture (IA), or by using microfluidics (MF) [27]. In dUC particle separation and extraction is achieved through molecular segregation according to size and buoyant density, allowing for extracting from large volumes of biological fluids with minimal use of reagents. Although this remains the most popular method, one major disadvantage is a consequential extraction on unwanted non-exosomal particles. SEC allows for the separation of molecules with different hydrodynamic radii and is frequently used to separate biopolymers. This approach is effectively used in difficult-to-extract EVs from blood plasma, urine protein complexes, and lipoproteins. However, this method is hampered by a relatively low EV yield and the need for expensive chromatographic sorbents. Similar to dUC, UF also separates EVs according to size and density but through filtering with membrane filters. UF allows for a purer extraction and is often used conjunctively with dUC and SEC. Hydrophilic polymers are used in PEG to extract EVs by exploiting differences in surface charge and solubility. This results in a method which is easily reproducible and scalable, however, is associated with a degree of sample contamination. In IA, antibodies against EV receptors enable a faster and purer isolation, but is let down by the need for expensive antibody products and difficulty in dividing EVs from antibody complexes. This produces a sample that is more unsatisfactory when compared to other methods due to poor functionality of EVs, and poor scalability. MF utilises devices consisting of small units having a network of microchannels of varying widths that can handle viscous fluids. Devices can extract EVs through immunoaffinity, size and density; with the most common being the immuno-microfluidic technique. It is similar to IA but allows for processing a much smaller sample for extraction and thus a decreased requirement of expensive antibody reagent [27, 28].

## Musculoskeletal (MSK) system

The lack of blood supply to articular cartilage makes osteoarthritis problematic to manage indefinitely. Recently, both BM-MSCs and adipose tissue-derived MSCs (AT-MSCs) have been used in the treatment of osteoarthritis (OA) [29–32]. However, there are still many disadvantages of using MSCs in these settings, including tumour formation [33]. Consequentially, novel research into iMSCs-derived exosomes has been undertaken to overcome these obstacles when treating OA.

A common issue associated with exosome therapy is the ideal cell source for the generation of exosomes [34, 35]. Synovial membrane-derived MSCs (SM-MSCs) may be a suitable exosome source for cartilaginous repair, as the synovium and cartilage are developed from a common pool of cells [36, 37]. This is supported by animal models where SM-MSCs inhibit OA progression [38, 39]. However, although SM-MSCs have proved to undergo chondrogenesis far more readily than alternatives such as BM-MSCs and AT-MSCs [40], SM-MSCs are notoriously hard to obtain and can only be acquired through an invasive approach [16]. An alternative source of MSCs are iMSCs. iMSCs in OA therapy carry many benefits, including self-renewal and vast differentiation capacity, as iPSCs have a similar morphology to embryonic stem cells (ESCs) but without ethical issue [15].

Zhu et al. conducted a study comparing the exosomes secreted by iMSCs and SM-MSCs in OA management [16]. Both exosomes were approximately 50–150 nm in diameter and expressed CD9, CD63, and TSG101. Both exosomes improved the OA in the mouse OA model; however, the iMSC-derived exosome therapy was superior to the SM-MSC-derived exosomes. Histologically, a reduction in Safranin O staining was found in the SM-SMC-exosome group compared to the iMSC-exosome group. This indicates a reduction in the loss of proteoglycan in cartilage in the iMSC-exosome group. Additionally, on immunohistochemistry analysis, the iMSC-exosome group had more Collagen II staining in the superficial zone of cartilage compared to the SM-MSC-exosome group. There were no significant differences noted in Collagen II staining in the deep zone and Collagen I staining between the two groups. This seemed to be correlated to a more robust chondrocyte migration in the iMSC-derived exosome therapy condition compared to the SM-MSC-derived exosome therapy [16].

Osteoporosis is another common MSK condition, primarily associated with old age. BM-MSC-derived exosomes from ovariectomised rats were found to increase osteoblast proliferation and alkaline phosphatase (ALP) activity and upregulate the expression of osteoblastic-related genes [41]. Furthermore, BM-MSC-derived exosomes have been shown to positively affect osteogenesis both *In vivo* and *In vitro*, and miR-196a carried in these exosomes was crucial to the positive regulation of the osteogenic genes while not inhibiting cellular proliferation [42]. Consequently, it can be concluded that MSC-derived exosomes have shown excellent prospects in treating osteoporosis [41, 42]. iMSC-exosomes in rat models have shown significant effects on preventing bone loss and promoting osteo-regeneration [41, 43, 44]. To replicate pathological osteoporosis the models incorporate a mix of calvarial defects of the ovariectomised rat osteoporosis models, engineered rat tissue and steroid-induced osteonecrosis of the femoral head [41, 43, 44]. Two studies reported that the PI3/Akt signalling pathway was critical for [44] the exosomes-mediated osteogenic and angiogenesis properties [43, 44]. Zhang et al. reported that tricalcium phosphate (TCP) combined with iMSC-derived exosomes resulted in significantly increased activation of the PI3/Akt signalling pathway and osteogenesis-related marker proteins such as Runx2, COL1 and OCN. Administration of PI3K inhibitor LY294002 markedly suppressed these osteogenic markers [43]. Furthermore, iMSC-derived exosomes prevented osteonecrosis of the femoral head by increasing the microvessel density in the femoral head [44]. The PI3/Akt pathway has also been crucial in promoting angiogenesis in that study [44].

**Fig. 1:**
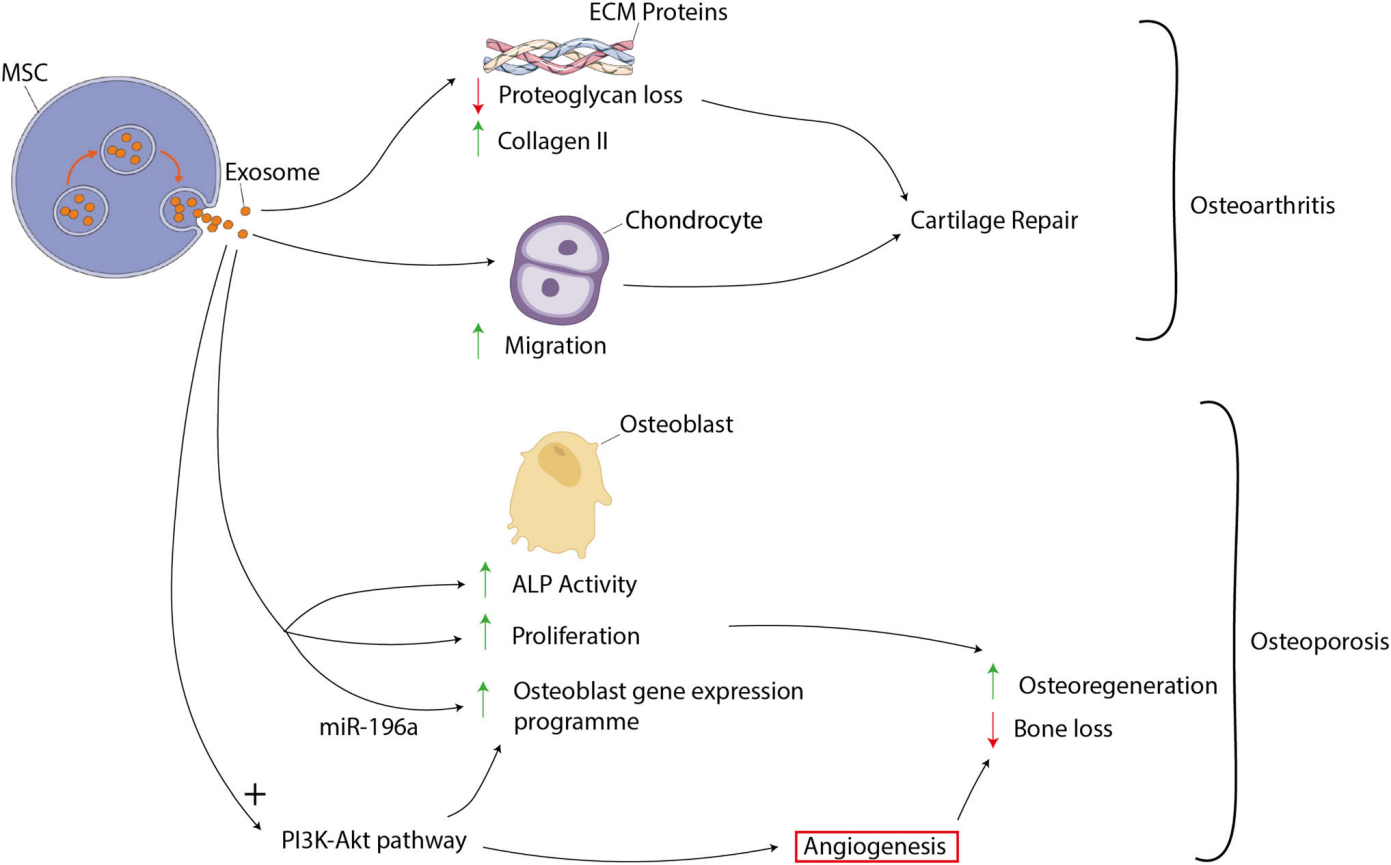
Mechanisms of exosome therapeutic efficacy in osteoarthritis and osteoporosis in osteoarthritis, MSC-derived exosomes promote cartilage repair. More specifically exosomes drive the increased deposition of type II collagen and reduced loss of proteoglycans within the cartilage extracellular matrix—this may potentially be due to exosomes driving chondrocyte migration. In osteoporosis, exosome-derived miR-196a promotes osteoregeneration activity within osteoblasts by increasing the expression of the osteoblast gene expression programme; the independent activation of the PI3K-Akt pathways by exosomes also promotes these gene expression changes. Furthermore exosomes augment osteoblast activity by increasing osteoblast proliferation and ALP activity. Independently, the activation of the PI3k-Akt pathway drives angiogenesis within the bone to promote healing. All together, through these actions on osteoblasts and angiogenesis, MSC-derived exosomes drive osteoregeneration and decreased bone loss in osteoporosis. Figure created using BioRender.com and Servier Medical Art templates, licensed under a Creative Commons Attribution 3.0 Unported License

Overall, the osteogenic properties of iMSC-exosomes are largely dependent on the activation of the PI3/Akt signalling pathway and future work in this field should focus on understanding the molecular contents of iMSC-exosomes (Fig. 1). iMSC-derived exosomes are theoretically inexhaustible while possessing many other benefits compared to alternatives; iMSC-derived exosomes could be a novel therapeutic approach for treating MSK pathologies, such as OA and osteoporosis.

## Cardiovascular disease

Cardiovascular disease includes pathologies from both the heart and blood vessels [45]. In this review, we focused mainly on cardiac pathologies as the evidence supporting iMSC-derived exosome therapy on limb ischaemia is limited; however, we have included the limb ischaemia studies in Table 1.

**Fig. 2:**
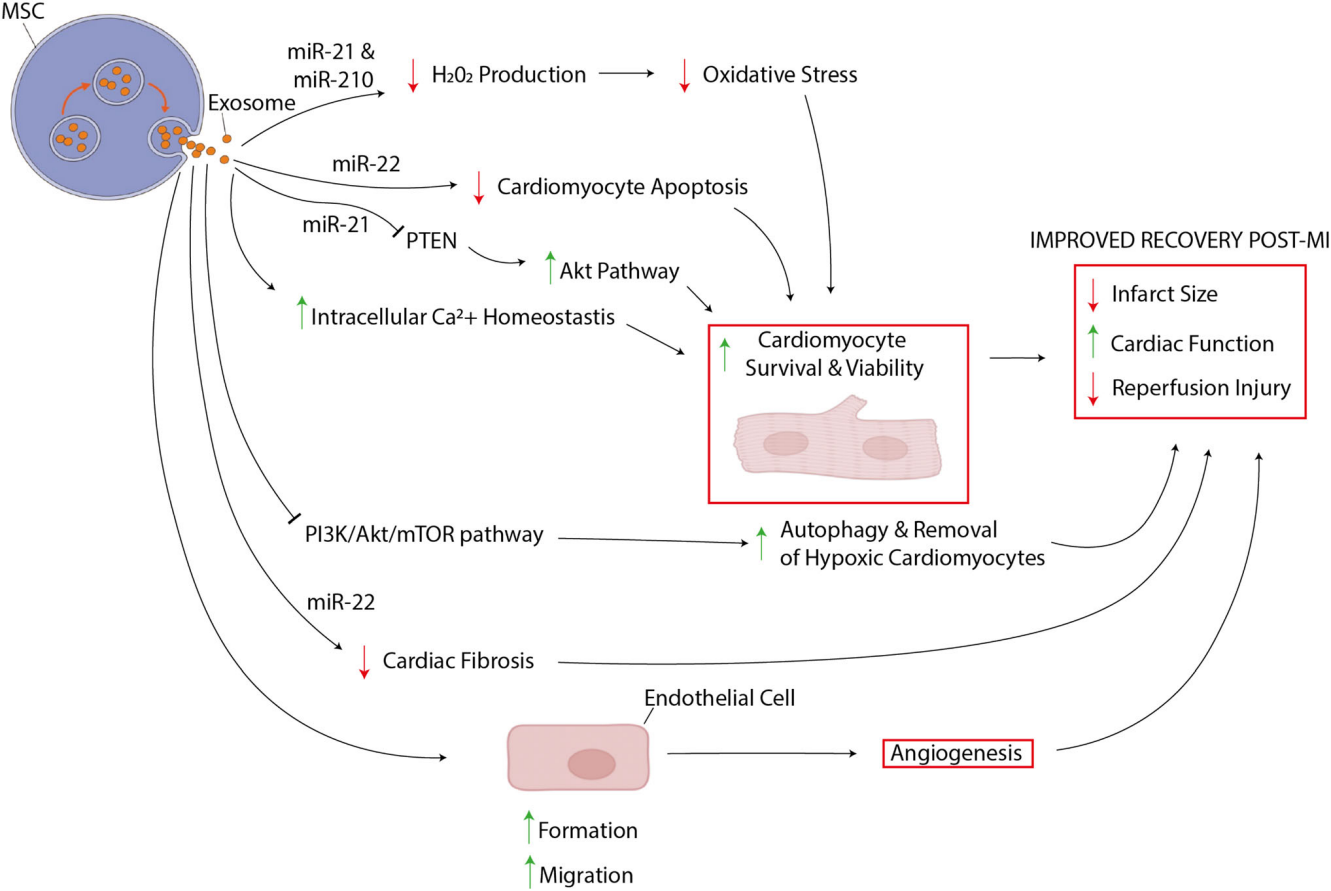
Therapeutic mechanisms of MSC-derived exosomes in myocardial infarction and reperfusion injury MSC-derived exosomes promote improved recovery following hypoxic cardiac injury through a variety of mechanisms. Broadly these mechanisms act on three areas to reduce injury following infarction and reperfusion (i) increasing cardiomyocyte survival and viability; (ii) decreasing fibrosis within the cardiac extracellular matrix; and (iii) promoting endothelial cells to develop new blood vessels (angiogenesis). MSC-derived exosomes promotes cardiomyocyte survival through several pathways: (ia) exosome-derived miR-21 and miR-210 inhibit hydrogen peroxide generation and thus suppress oxidative stress; (ib) miR-22 within exosomes inhibits apoptotic programmes; (ic) exosomes containing miR-21 inhibit PTEN and thus increase the activity of the pro-survival Akt pathway; and (iv) exosomes promote homeostasis of intracellular calcium stores to maintain cardiomyocyte excitation–contraction coupling and contractile efficiency. Alongside this, exosome-contents inhibit the PI3K/Akt/mTOR pathway which increases autophagy and removal of hypoxic and injured cardiomyocytes that would otherwise cause inefficient contraction. Focusing now on the ECM, miR-22 inhibits cardiac fibrosis which preserves cardiac contractility and output. Moreover, exosomes promote angiogenesis at sites of cardiac injury by driving the migration and generation of endothelial cells, which may contribute to improved recovery. Figure created using BioRender.com and Servier Medical Art templates, licensed under a Creative Commons Attribution 3.0 Unported License

MSC-derived exosomes have been found to play an essential role in reperfusion injury (Fig. 2). A study demonstrated that BM-MSC-derived exosomes maintain the systolic and diastolic contractility of the myocardium while reducing the infarct size [46]. Furthermore, AT-MSC-derived exosome therapy has been shown to increase cell viability under hypoxia *In vitro* and could protect against reperfusion injury during myocardial ischaemia *In vivo* through the activation Wnt/*β*-catenin signalling [47]. Human ESC-MSC-derived exosomes therapy led to a 45% reduction in infarct size in a mouse reperfusion injury model compared to the saline control [24]. In addition, following exosome treatment, there were increased levels of ATP, NADH, phosphorylated Akt, and phosphorylated GSK-3*β*, while the levels of oxidative stress and phosphorylated-JNK decreased [48]. Arslan et al. also demonstrated a significant increase in reperfusion post-myocardial ischaemia following intramyocardial injection of ESC-MSC-derived exosomes [48].

As well as playing a quintessential role in reducing reperfusion injury, MSC-derived exosomes enriched with miR-22 demonstrate cardioprotective properties by reducing cardiac fibrosis and preventing further apoptosis [49]. Other studies have identified other microRNAs, which also play a role in cardioprotection [50]. miR-21 enriched MSC-derived exosomes can suppress the expression of PTEN in cardiomyocytes, which resulted in the activation of the Akt signalling pathway, contributing to increased cell survival [50].

There is a strong case for MSC-derived exosomes in reducing cardiovascular disease, as this has been confirmed by a meta-analysis specifically dedicated to MSC-derived exosomes reducing myocardial reperfusion injury [51]. Although there is far less evidence specific to iMSC-derived exosome therapy in cardiovascular disease, the results are promising [52–54]. Intramyocardial injection of mouse iMSC-derived exosomes was found to be cardioprotective during reperfusion injury [54]. *In vitro*, Nanog-regulated miR-21 and hypoxia-inducible factor 1 regulated miR-210 were found to inhibit caspase 3/7 activation, which was responsible for preventing hydrogen peroxide (H_2_O_2_)-induced oxidative stress on cardiomyocytes during myocardial ischaemia [54].

Furthermore, another study using a swine myocardial ischaemia model also found similar cardioprotective effects [53]. *In vivo,* iPSC-derived cardiomyocyte exosomes significantly improved physiological cardiac functions such as left ventricular ejection fraction, wall stress, and cardiac hypertrophy while improving angiogenesis in the infarct and decreasing scar size and myocardial apoptosis [53]. Mechanistically, iPSC-derived cardiomyocyte exosomes increased endothelial cell formation and angiogenesis while reducing apoptosis and maintaining intracellular calcium homeostasis to promote a healthy cardiac microenvironment [53]. iPSC-derived cardiomyocyte exosome therapy carries many cardiac benefits and was not found to increase the frequency of arrhythmias compared to the control [53]. In addition, Santoso et al. showed that iPSC-derived cardiomyocyte exosome therapy could be used to promote autophagy of hypoxic cardiomyocytes to facilitate recovery post-myocardial infarction [52]. Further gene profiling in that study revealed that the inhibition of the PI3K‐Akt‐mTOR signalling pathway was responsible for enhanced autophagy and improved physiological cardiac recovery [52]. To summarise, iMSC-derived exosomes and iPSC-derived exosomes have massive potential to improve recovery in post-myocardial infarction patients through many mechanisms. In addition, the therapeutic effects can be achieved without increasing the frequency of arrhythmogenic complications [53] thus providing a promising therapeutic option for myocardial injury.

The research on exosomes in cardiovascular disease is currently moving towards identifying future microRNA candidates to be incorporated and enriched in iMSC-derived exosomes.

## Wound healing

A wound is defined as a disruption in skin integrity, mucous membranes and organ tissues [55]. A systematic review encompassing over 313 studies which used both BM-MSC and AT-MSC in wound healing demonstrated that healing in diabetic wounds was significantly enhanced with MSC-derived exosome therapy, compared to the control cohorts with a standard mean deviation of 5.48 at a 95% confidence interval [56]. However, to our knowledge, there is no meta-analysis to date that synthesises the therapeutic effects of MSC-derived exosomes in wound healing outside diabetic wounds. Thus, further research in this field can dramatically enhance our understanding of EVs in wound healing.

As mentioned earlier, iMSCs hold tremendous advantages compared to adult MSC-derived therapy. Nevertheless, there is doubt on whether iMSC-derived exosomes can promote skin growth in the same way as adult MSC-derived therapy. Excitingly, a recent study from Zhang et al. showed that iMSC-derived exosomes promoted wound healing via enhancing collagen synthesis and angiogenesis through the stimulation of human dermal fibroblasts (HDFs) and human umbilical vein endothelial cells (HUVECs) [25]. Furthermore, iMSC-derived exosome therapy in diabetic ulcer mice demonstrated faster wound healing and closure rate [57]. This was explained by Kobayashi et al.’s *In vitro* study; on the scratch assay, iMSC-derived exosomes treated fibroblasts had far greater migratory ability compared to MSCs-derived exosome treated fibroblasts [57].

An *In vitro* study compared the ability of MSC-derived exosomes and iMSC-derived exosomes in promoting skin cell proliferation [58]. The wound scratch assay demonstrated that iMSC-derived exosomes are superior to adult MSC-derived exosomes, showcased by iMSC-derived exosomes leading to significantly enhanced growth rate of human keratinocytes compared to adult MSC-derived exosomes therapy, and this correlated to the smaller wound area after both 24 h and 48 h post-therapy [58]. Studies also found that the induced proliferation of skin cells by both adult MSC and iMSC-derived exosome therapies were mediated by the ERK1/2 pathway [59–61]. This suggests mechanistic pathway conservation in the activation of skin cells between adult MSC and iMSC-derived exosomes and that MSC-exosomes therapy can be reproduced by iMSC-derived therapy, with the additional benefits of iMSCs. On the other hand, others have found that a significant increase in ERK1/2 phosphorylation in keratinocytes was observed after iMSC-derived exosome therapy; but adult MSC-derived exosomes failed to show the same effects [58]. The differential effects of iMSCs and adult MSCs exosomes are thought to be due to distinct biological cargos, for example, surface mitogens and mRNA epigenetics [58].

**Fig. 3:**
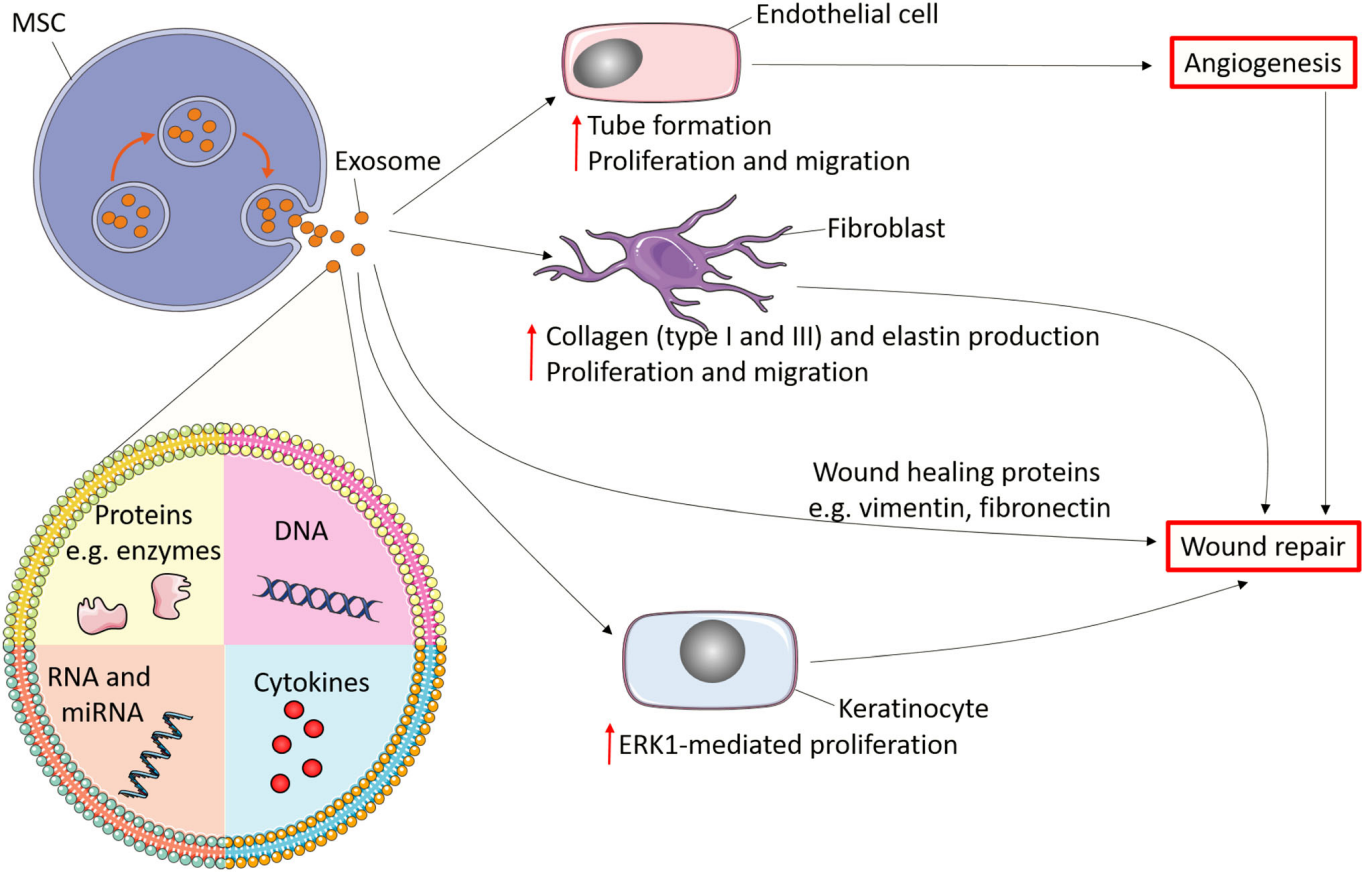
Mechanisms of wound healing by MSC-derived exosomes MSC-derived exosomes promote wound healing through multiple mechanisms. Briefly, exosomes can enhance fibroblast production of type I and III collagen and elastin, and promote fibroblast proliferation and migration which contributes to wound healing. Furthermore, exosomes contain a multitude of wound-healing proteins including vimentin, ankyrin, fibrillin, desmin and fibronectin which can directly induce wound healing. Exosomes also increase phosphorylated ERK1 in keratinocytes leading to epithelisation hence wound regeneration. Finally, exosomes can also promote wound repair by inducing angiogenesis via stimulating endothelial cell proliferation, migration and tube formation in a PKA/VEGF pathway dependant manner [16, 25, 57, 58, 62, 63, 65]. Figure created using Servier Medical Art templates, licensed under a Creative Commons Attribution 3.0 Unported License

Increasing studies begin to characterise the mechanisms behind wound healing by iMSC-derived exosome therapy (Fig. 3). To further our understanding of MSC-derived exosomes, we need to explore the extrinsic environments which may promote the function of MSC-derived exosomes. For example, acellular Wharton's jelly embedded with MSCs produced exosomes that contained various wound-healing proteins such as vimentin, ankyrin, fibrillin, desmin and fibronectin [62, 63]. The study demonstrates that the exosomes from Wharton’s jelly tissue directly contributed to wound-healing [62]; therefore, futures avenues of research should ideally compare the biomechanical composition of MSC and iMSC-derived exosomes extracted from acellular Wharton’s jelly to further our mechanistic understanding.

To assess the potential therapeutic effects of iPSC-derived exosome therapy in humans, a rhesus macaque wound healing model was used to assess the differences in autologous and allogenic iPSC-derived exosome therapy [64]. Both groups demonstrated accelerated wound healing, epithelial coverage, collagen deposition, and angiogenesis; however, the autologous iPSC-derived exosomes therapy was more effective than their allogenic counterparts [64]. There were significantly more exosomes present in the wound in autologous transplants than the allogenic counterpart; this is likely to have contributed to the autologous iPSC-derived exosome therapy more effectively promoting wound healing, epithelisation, and angiogenesis [64]. Although autologous iPSC-exosomes therapy is more effective than the allogenic counterpart, allogenic iPSC-exosome therapy should be the preferred choice for “off-the-shelf” iPSC-exosomes therapy. Importantly, all autologous iPSCs treated rhesus macaques developed teratomas, whereas none of the allogenic counterparts developed this complication [64, 65]. This is likely due to the implantation of the iPSCs as the exosomes derived from the iPSCs do not contain any chromosomes; thus unlikely to be the cause of the teratoma.

## Neurology: neurodegeneration and corneal defects

**Fig. 4:**
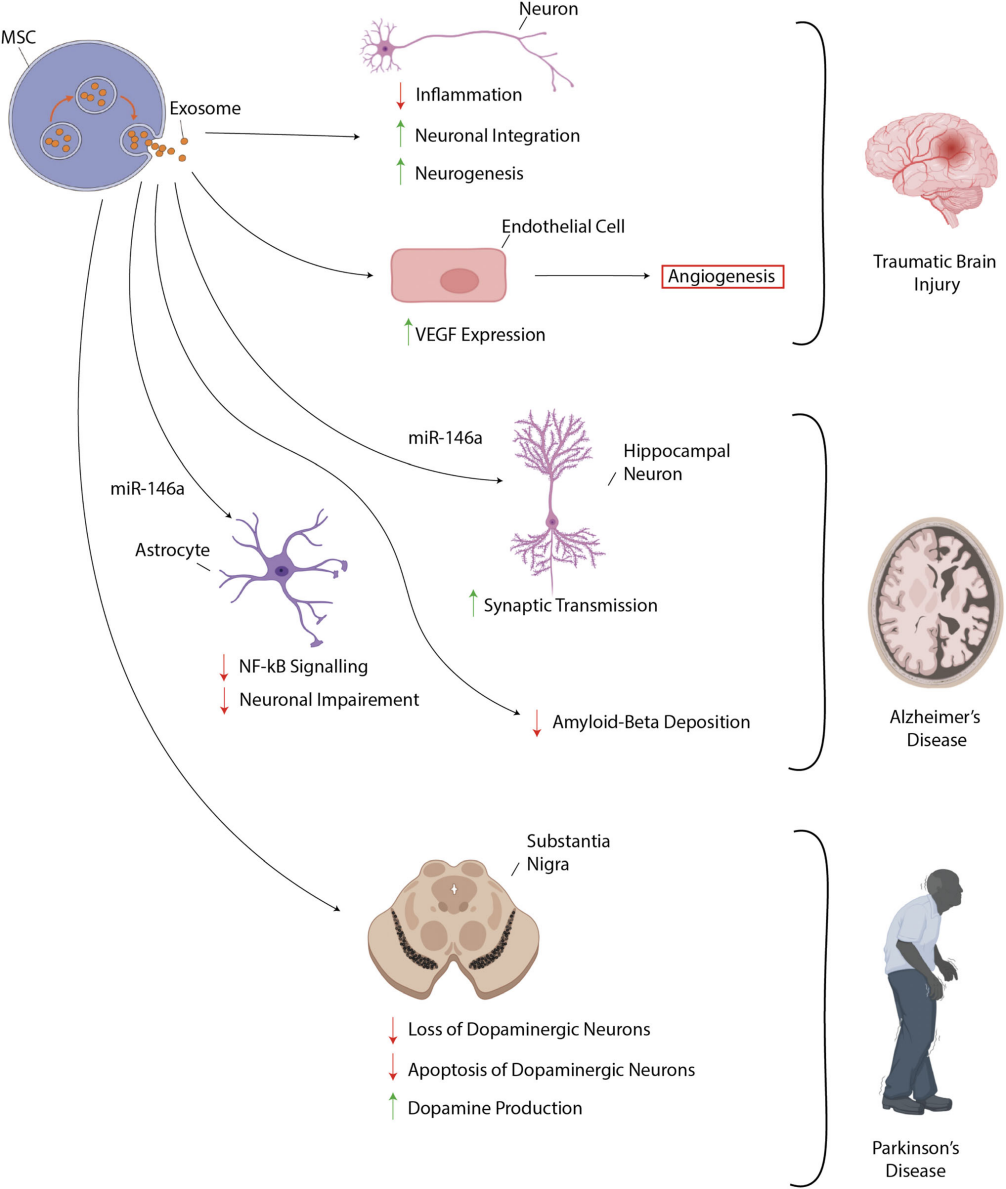
Therapeutic efficacy of MSC-derived exosomes in traumatic brain injury (TBI), Alzheimer’s disease and Parkinson’s disease MSC-derived exosomes have shown therapeutic efficacy in several neurological diseases. Following TBI exosomes have shown to promote the generation of new neurons, increase neuronal integration and reduce inflammation. In the context of TBI, exosomes also augment VEGF expression in endothelial cells to drive angiogenesis which may potentially improve tissue recovery by promoting increased blood flow. In the context of Alzheimer's disease, exosome-derived miR-146a increases synaptic transmission in hippocampal neurons as well as diminishing NF-kB induced neuronal impairment in astrocytes. Exosomes also decrease amyloid-beta deposition. In the context of Parkinson’s disease MSC-derived exosomes act at the substantia nigra to increase dopamine production and decrease apoptosis and loss of dopaminergic neurons. Figure created using BioRender.com and Servier Medical Art templates, licensed under a Creative Commons Attribution 3.0 Unported License

The therapeutic potential of MSC-derived exosome therapy in neurodegenerative diseases is unclear [65, 66]. Although current evidence is sparse, a growing number of studies have begun to demonstrate the high potential of MSC-derived exosomes in the treatment of neurodegenerative disease [67]. A recent report tested whether systemic administration of MSC-derived exosomes promotes functional recovery and neurovascular remodelling in rats after traumatic brain injury (TBI) [68]. The study demonstrates that MSCs-derived exosomes significantly improved the Morris water maze test results and sensorimotor function compared to the saline-treated controls [68]. On histological analysis, animals that received the MSC-derived exosome therapy had significantly increased numbers of newly generated endothelial cells in the TBI lesion boundary, integration of mature and immature neurons in the dentate gyrus, and simultaneously reducing inflammation in the dentate gyrus [68]. This suggests that MSC-derived exosome therapy can improve functional recovery by promoting endogenous angiogenesis, neurogenesis, and reducing inflammation. The underlying mechanism of exosomes on the functional recovery post-TBI is unclear (Fig. 4). However, a recent study alluded to the signalling pathway being crucial in promoting VEGF expression and could be vital in uncovering safe and effective treatments for neurodegenerative diseases [65].

The overwhelming majority of all potential drugs beneficial for the central nervous system are not used clinically because of their inability to penetrate the blood–brain barrier (BBB), with around 98% of small molecules unable to cross the BBB [69]. Despite this, there is still a lot of promise for MSC-exosome therapy in neurodegenerative diseases such as Parkinson’s disease and Alzheimer’s disease [70–72]. BM-MSCs exosome therapy has been tested in mouse models of Alzheimer's disease and was found to reduce neural impairment and improve synaptic transmission in the Alzheimer’s disease rat model hippocampi [71]. BM-MSC-derived exosomes increased the expression of miRNA-146a in the hippocampi while simultaneously decreasing the levels of nuclear factor kappa B (NF-κB) in astrocytes which in turn resulted in synaptogenesis and the amelioration of neural impairment [71]. The therapeutic effects of MSCs-derived exosomes are not limited to BM-MSCs; a study using human umbilical cord derived MSC (hUC-MSC)-derived exosomes in a mouse model of Alzheimer’s disease found similar benefits and improved cognitive dysfunction and cleared A*β* deposition [72]. hUC-MSCs are beneficial in Parkinson’s disease as well. Chen et al. demonstrated that hUC-MSC-derived exosomes can reach the substantia nigra, penetrating through the BBB *In vivo* [70]. The study found that the huC-MSC-derived exosomes reduced the loss of dopaminergic neurons and apoptosis while increasing dopamine levels in the basal ganglia [70].

In the literature, the focus of iPSC-derived-exosomes therapy is Alzheimer’s disease (AD); the defining hallmarks of AD are the progressive accumulation of amyloid-*β* (A*β*) and hyperphosphorylated tau proteins [73, 74]. Instead of inhibiting the progression of Alzheimer’s disease, the literature discusses the potential propagation of tau pathology *In vivo* in mouse models [75, 76]. Both studies found that the neuronally differentiated iPSC-derived exosomes that express the tau P301L and V337M domains resulted in the presence of hyperphosphorylated tau inclusion throughout the brain and extensive degeneration of neuronal dendrites in both hippocampi [75, 76]. Thus, these results indicate that exosomes are sufficient to allow for *In vivo* propagation of tau pathology in mouse models [76]. Another study demonstrated that miR-137 and the expression of the CACNAC1 gene could inhibit the hyperphosphorylation of tau proteins and inhibit the progression of Alzheimer’s disease [77]. Thus, if miR-137 could be integrated into an exosome, iPSC-derived exosome therapy can inhibit the propagation of tau pathology and, in turn, halt the progression of Alzheimer’s disease.

Sharma et al. demonstrated that exosomes could regulate the development of neural circuits [78]. Proteomic and bioinformatic analysis revealed that treating the iPSC-derived cultures lacking methyl-CpG binding protein 2 (MECP2) with exosomes containing MECP2 rescued the deficits in neuronal proliferation, differentiation and synaptogenesis [78]. However, those MECP2-deficient cultures lacked the same neurophysiological capacities [78].

Ophthalmological pathology is closely related to neurological conditions and can facilitate a bidirectional relationship [79–81]. Corneal epithelial defects are particularly worrying as they render the eye susceptible to infection, scarring, perforation and ulceration, leading to vision loss [82, 83]. Wang et al. demonstrated that both iMSC and MSC exosome therapies accelerate corneal defect healing *In vivo*, and iMSC-derived exosome therapy was more effective than the MSC counterpart [84]. *In vitro,* iMSC exosome therapy exhibited a more substantial effect on proliferation, migration, cell cycle promotion and apoptosis inhibition of corneal epithelial cells [84]. Although the therapeutic effects of iMSC-derived exosome therapy was more potent than its counterpart, both exosome therapies upregulated CDK2 and cyclin A to catalyse corneal epithelial cells to enter the S phase of the cell cycle [84].

## Fibrosis

Fibrosis is the abnormal formation of scarring due to excess deposition of the extracellular matrix [85]. This permanent scarring in internal organs commonly leads to organ failure and ultimately death, as seen in cirrhosis, end-stage kidney disease and idiopathic pulmonary fibrosis [86, 87]. Therefore, it is imperative to discover a way to control fibrosis to improve patients’ quality of life with end-stage organ failure. BM-MSC-derived exosomes have already been shown to ameliorate liver fibrosis and kidney fibrosis [88–90]. However, the potential of iMSC-derived exosomes in this field is still relatively unknown.

In one study, iMSC-derived exosomes were tested on a unilateral ureteral obstruction (UUO) mouse model and found to reduce renal fibrosis and improve renal function [91]. The reduction in renal fibrosis was correlated to increasing exosome concentrations and reduced differentiation of NRK-52E cells [91]. The anti-fibrotic effects of iMSC-derived exosomes are not limited to the kidney. Another study used a murine liver ischaemia/reperfusion model where the iMSC-derived exosomes were administered via the inferior vena cava [92]. The therapeutic effects of the iMSC-derived exosomes included a reduction in histopathological signatures of liver fibrosis (hepatocyte necrosis and sinusoidal congestion), reduction in serum aspartate aminotransferase (AST) and alanine aminotransferase (ALT) and promoted hepatocyte proliferation in a dose-dependent manner [92]. Du et al. found that the exosomes activated sphingosine kinase (SK1) and sphingosine-1-phosphate (S1P1) in hepatocytes to promote cellular proliferation [92]. Nong et al. demonstrated the same hepatoprotective effects as Du et al. in a rat liver ischaemia/reperfusion injury model [92, 93]. However, Nong et al. concluded an alternative mechanism of action; via suppression of inflammatory mediators (tumour necrosis factor-alpha and interleukin-6 (IL-6)), apoptosis (caspase-3 and bax) and oxidation (glutathione, glutathione peroxidase and superoxide dismutase) [93]. IL-6 contributes to Th17 induction which has important roles in the pathogenesis of organ fibrosis [94], and hence suppression of IL-6 may reduce fibrosis (Fig. 2). These findings represent a novel therapeutic approach to tackling fibrosis in end-stage organ failure patients to improve the quality of life.

## Graft vs host disease (GvHD)

Donated bone marrow/stem cells may be viewed by the recipient’s body as foreign, and the donated cells mount an attack on the host known as GvHD [95]. MSC has been introduced as a treatment method for GvHD and this can be attributed to the immunosuppressive nature of MSCs [96, 97]. MSC-derived exosomes have the same immunosuppressive effects and can be applied to GvHD [98, 99]. A study published in 2014 described the first successful treatment of GvHD in humans with MSC-derived exosomes [100]. The patient was stable for several months and died of an unrelated cause [100]. This study demonstrates high potential for using MSC-derived exosomes in GvHD; however, there is still little known regarding the method’s efficiency.

**Fig. 5:**
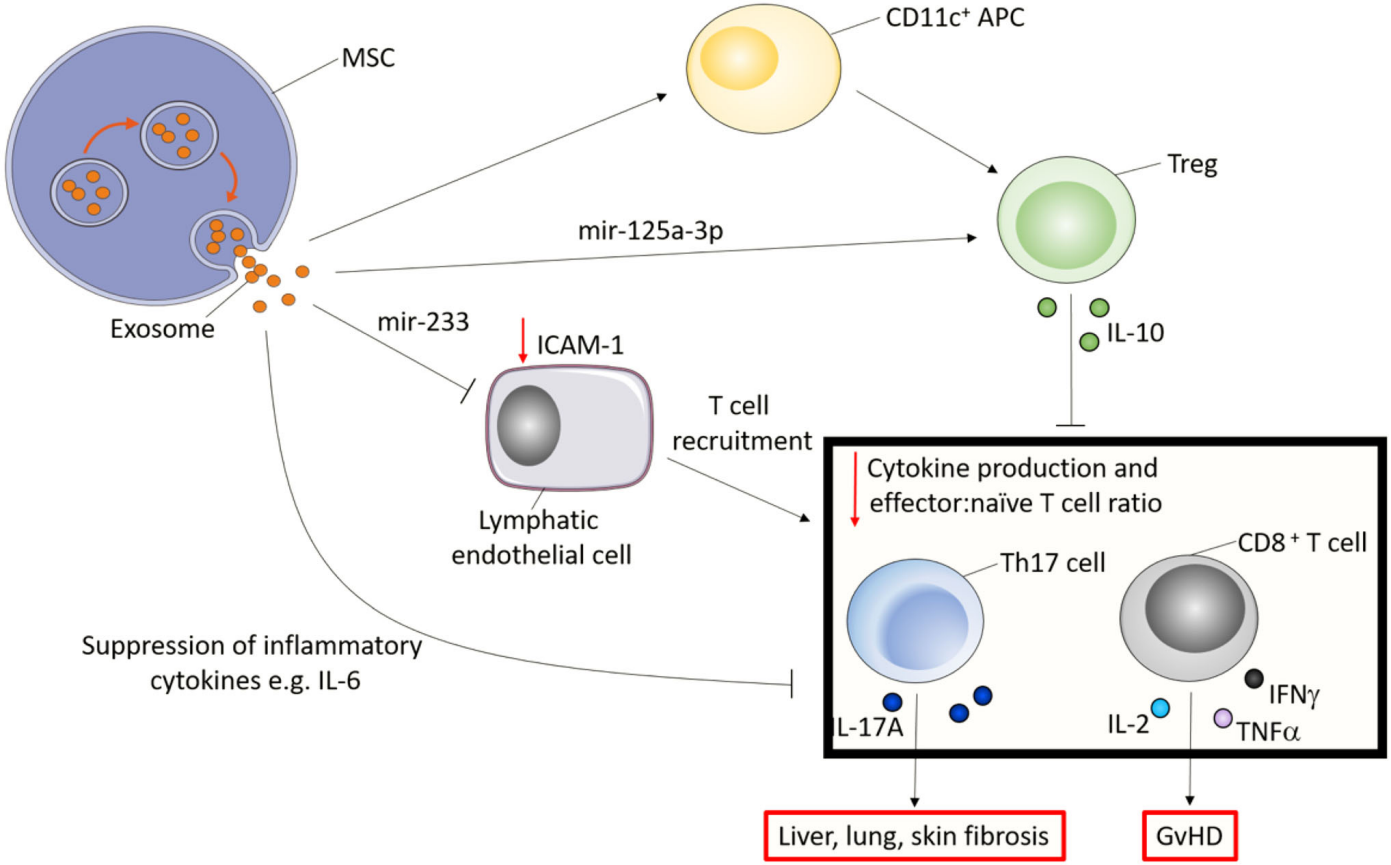
Potential therapeutic efficacy of MSC-derived exosomes in preventing immune-mediated organ fibrosis and GvHD. Th17 cells are important in the pathogenesis of liver, lung and skin fibrosis, while CD8^+^ T cells are potent drivers are graft versus host disease (GvHD). MSC-derived exosomes promote CD11c^+^ antigen-presenting cells (APC) to induce Tregs, which secretes the anti-inflammatory cytokine IL-10 to suppress Th17 and CD8^+^ T cells. Furthermore, exosomes contain mir-125a-3p which may also contribute to Treg induction. Treg-mediated suppression results in reduced pro-inflammatory IL-17A production by Th17 cells, and IL-2, TNF *α* and IFN*γ* by CD8^+^ T cells. Furthermore, exosome-derived mir-233 decreases ICAM-1 expression on lymphatic endothelial cells, thereby decreasing T cell adhesion and migration. Finally, exosomes can also exert direct anti-inflammatory effects, for example in reducing IL-6, thereby decreasing Th17 polarisation and T cell activation and hence reducing immune-mediated pathologies including organ fibrosis and GvHD [93, 94,101–105]. Figure created using Servier Medical Art templates, licensed under a Creative Commons Attribution 3.0 Unported License

Growing studies begin to uncover the mechanisms behind MSC-derived exosomes in preventing GvHD (Fig. 5). Injection of MSC-derived exosomes into a chronic GvHD mouse model via the tail vein found that MSC-derived exosomes improved survival and pathological damage from chronic GvHD by suppressing Th17 and inducing T_reg_ cells [101]. Other studies have demonstrated similar suppressive findings and further suggest that MSC-derived exosomes induce T_reg_ cells through the APC-mediated pathway [102]. Unfortunately, there is yet to be a study published discussing the use of iMSC-derived exosomes in GvHD. However, given the success of MSC-derived exosomes and the added value of iMSC-derived exosomes, it may be speculated that iMSC-derived exosomes can be an effective and feasible treatment method for GvHD.

## Clinical limitations

iMSC-derived exosomes are a relatively modern innovation, with the first in-vitro studies being conducted under a decade ago [106]. As evidenced in this paper, the prospect of using iMSC exosomes in certain clinical conditions is promising, however due to time alone there is a limited quantity of evidence for each clinical application, particularly in-vitro, and none at a clinical trial level. Historically, there have only been 26 clinical trials worldwide that utilised MSC-derived exosome therapy, however none of the stem cells used were generated from iPSC’s [109]. Out of the 26 clinical trials on the topic of MSC-exosomes, 12 of them have been designed with the purpose of determining the safety and tolerability of MSC-exosomes. It is a possibility that in larger in-vitro or human sample sizes, unforeseen complications arise, related to the complexity of accurately administering treatment in heterogenous subgroups of patients, as well as safety and clinical efficacy.

One of the key safety concerns regarding MSCs, is their immunogenicity. As the cells are allogeneic, there is an increased risk of a stimulated immune reaction when transplanted to their donor. Use of exosomes reduce this risk greatly, however using products generated from cells that may not be immunocompatible with the donor, has the potential to also cause harm. Out of the 26 clinical trials on the topic of MSC-exosomes, 12 of them have been designed with the purpose of determining the safety and tolerability of MSC-exosomes, meaning novel data on immunogenicity should be available in the near future.

Other potential disadvantages and concerns regarding iMSC-derived Exosomes that have been detailed in the literature include:

1. Manufacturing Inefficiencies–current exosomal extraction techniques from iMSCs are met with the compromise of either high costs, low yield, or impurity. [27, 28]
2. Non-Standardised Manufacturing–small differences in different isolation and purification methods result in a variety of exosomal content, characteristics, and function. Precise individualised distillation techniques must be developed for specific clinical applications, so that the extracted exosomes possess the characteristics for intended use. [110]
3. Induction of Cells alters Exosome Content–during the induction process, PSCs are exposed to various transcriptional and epigenetic factors for them to differentiate into MSCs. Further studies into how these factors affect the contents produced by the exosomes, would be heavily valued. [111]
4. Complexity of Manufacturing—Differentiation of PSCs into MSCs adds an extra layer of complexity when it comes to determining the most efficient, cost-effective, and safe way to generate the cell line, that will in turn also produce exosomes of a desirable quality. [18]

It is clear that we are in the infancy of research surrounding iMSC-derived exosomes, and that the main obstacles faced in escalating this, are those concerning the optimal conditions to manufacture the cells themselves, as well as the exosomes extracted. Concerns regarding clinical complications upon application of the exosomes are yet to be studied in depth.

## Limitations of review

We used a wide range of studies from the twentieth century onwards through a combination of MEDLINE, EMBASE and Cochrane. However, as many of the studies were not available on online publicly, we had to references search to find older papers. Although our process was rigorous, it is possible that there is missing literature on the topic.

## Conclusion

Current literature is based upon evidence from BM-MSCs or the umbilical cord and not iMSCs. Although the evidence is limited for the therapeutic effects of iMSC-derived exosomes, the current literature shows much promise as iMSCs resolve many of the problems associated with MSC derived exosome therapy. The more significant problems include immunosuppression and exhaustibility as current methods for large-scale MSCs face several obstacles because the amount of MSCs obtained from donors is insufficient [108]. However, there are still many uncertainties regarding iMSCs therapy, including selecting the optimal cell type for iMSCs generation because the differentiation potential of iMSCs can be affected by the origin of the donor cell [34, 35]. This would be an avenue for future research, and more studies will be required to optimise iMSC-derived exosome therapy. Although wound healing, cardiovascular disease and musculoskeletal pathology have the best prospects for iMSC-derived exosome therapy, further research is required to bridge our current understanding to clinical therapy. Additionally, rigorous testing of the iMSC and exosomal manufacturing processes should be conducted in future, to determine the most sustainable ways to produce reliable and effective product. An exciting field would be the use of iMSC-derived exosomes to treat GvHD, as current literature has shown successes with MSC-derived exosomes. In addition, the added benefits of iMSCs over MSCs may make future research and treatment more effective and feasible. A meta-analysis would be suitable for any of the topics mentioned above, especially on wound healing. In this short review, we have provided a clinical perspective on iMSC-derived exosome therapy to informs clinicians of pertinent information for future clinical stem cell therapies.
